# Immune-related gene signature to predict TACE refractoriness in patients with hepatocellular carcinoma based on artificial neural network

**DOI:** 10.3389/fgene.2022.993509

**Published:** 2023-01-04

**Authors:** Qingyu Xu, Chendong Wang, Guowen Yin

**Affiliations:** Department of Interventional Radiology, Jiangsu Cancer Hospital, Jiangsu Institute of Cancer Research, The Affiliated Cancer Hospital of Nanjing Medical University, Nanjing, China

**Keywords:** immune, signature, TACE, HCC, machine learning, artificial neural network

## Abstract

**Background:** Transarterial chemoembolization (TACE) is the standard treatment option for intermediate-stage hepatocellular carcinoma (HCC), while response varies among patients. This study aimed to identify novel immune-related genes (IRGs) and establish a prediction model for TACE refractoriness in HCC patients based on machine learning methods.

**Methods:** Gene expression data were downloaded from GSE104580 dataset of Gene Expression Omnibus (GEO) database, differential analysis was first performed to screen differentially expressed genes (DEGs). The least absolute shrinkage and selection operator (LASSO) regression analysis was performed to further select significant DEGs. Weighted gene co-expression network analysis (WGCNA) was utilized to build a gene co-expression network and filter the hub genes. Final signature genes were determined by the intersection of LASSO analysis results, WGCNA results and IRGs list. Based on the above results, the artificial neural network (ANN) model was constructed in the training cohort and verified in the validation cohort. Receiver operating characteristics (ROC) analysis was used to assess the prediction accuracy. Correlation of signature genes with tumor microenvironment scores, immune cells and immune checkpoint molecules were further analyzed. The tumor immune dysfunction and exclusion (TIDE) score was used to evaluate the response to immunotherapy.

**Results:** One hundred and forty-seven samples were included in this study, which was randomly divided into the training cohort (*n* = 103) and validation cohort (*n* = 44). In total, 224 genes were identified as DEGs. Further LASSO regression analysis screened out 25 genes from all DEGs. Through the intersection of LASSO results, WGCNA results and IRGs list, *S100A9*, *TREM1*, *COLEC12*, and *IFIT1* were integrated to construct the ANN model. The areas under the curves (AUCs) of the model were .887 in training cohort and .765 in validation cohort. The four IRGs also correlated with tumor microenvironment scores, infiltrated immune cells and immune checkpoint genes in various degrees. Patients with TACE-Response, lower expression of *COLEC12*, *S100A9*, *TREM1* and higher expression of *IFIT1* had better response to immunotherapy.

**Conclusion:** This study constructed and validated an IRG signature to predict the refractoriness to TACE in patients with HCC, which may have the potential to provide insights into the TACE refractoriness in HCC and become the immunotherapeutic targets for HCC patients with TACE refractoriness.

## Introduction

Primary liver cancer is the sixth most commonly diagnosed malignancies and the fourth leading cause of cancer-related death worldwide (Kelley and Greten, 2021). Hepatocellular carcinoma (HCC) is the most common histological type, accounting for 75%–95% of all primary liver cancer cases (Bray et al., 2018). However, nearly 70% of new patients are diagnosed at intermediate or advanced stage and miss the opportunity for curative resection (Song, 2015). Despite multimodal therapeutic advancements, the prognosis of HCC patients remains dismal with 5-years survival rate less than 20% (Marron et al., 2022).

Transarterial chemoembolization (TACE) is a standard treatment for intermediate-stage hepatocellular carcinoma (HCC) according to the Barcelona Clinic Liver Cancer (BCLC) staging system (Llovet et al., 1999; Han and Kim, 2015). TACE can be performed with conventional TACE (cTACE) and drug-eluting beads TACE (DEB-TACE). However, the response to TACE varies from different patients due to the heterogeneity in intermediate-stage HCC, and the concept of TACE refractoriness was subsequently introduced by several organizations (Park et al., 2013; Kudo et al., 2014; Raoul et al., 2014).

Although sorafenib therapy is recommended after TACE refractoriness based on the current TACE guidelines (Arizumi et al., 2015), other protocols including DEB-TACE, hepatic arterial infusion chemotherapy (HAIC), ablation, and TACE combined with systemic therapies are potentially effective as subsequent treatment after TACE refractoriness (Kobayashi et al., 2020; Zheng et al., 2020; Hsu et al., 2021; Kaibori et al., 2021). Currently, it is still lacking in the effective tools for predicting TACE response and selecting best candidates prior to TACE treatment.

In recent years, with the rapid progress in high-throughput profiling, the microarray technique has been extensively applied through identifying variant gene expression and pathways in considerable studies to disclose the molecular mechanisms of tumor onset and progression (Zhang et al., 2020; Wang et al., 2022a; Yang et al., 2022). Despite of its role in precision medicine, it is still characterized with higher costs and data inconsistency across different cohorts, also influenced by alignment or mapping method and the quantification model adopted. At present, machine learning algorithms have also been widely applied to screen disease-related genes and build prediction model (Peng et al., 2021; Han et al., 2022). RNA-seq data of HCC obtained from The Cancer Genome Atlas (TCGA) or Gene Expression Omnibus (GEO) databases have been utilized to identify genes associated with HCC carcinogenesis or prognosis, and elucidate the potential underlying mechanisms from various perspectives. To the best of our knowledge, no immune-related gene signature has been established for TACE refractoriness.

By using the bioinformatics analysis of GEO database, the aim of this study is to construct and validate the immune-related gene signature based on machine learning algorithms for predicting TACE refractoriness in patients with unresectable HCC. It is anticipated that this signature could help make decisions for TACE operations and provide novel insights into the understanding of mechanism of TACE refractoriness in HCC patients from the immunological perspective.

## Materials and methods

### Data acquisition and processing

**FIGURE 1:**
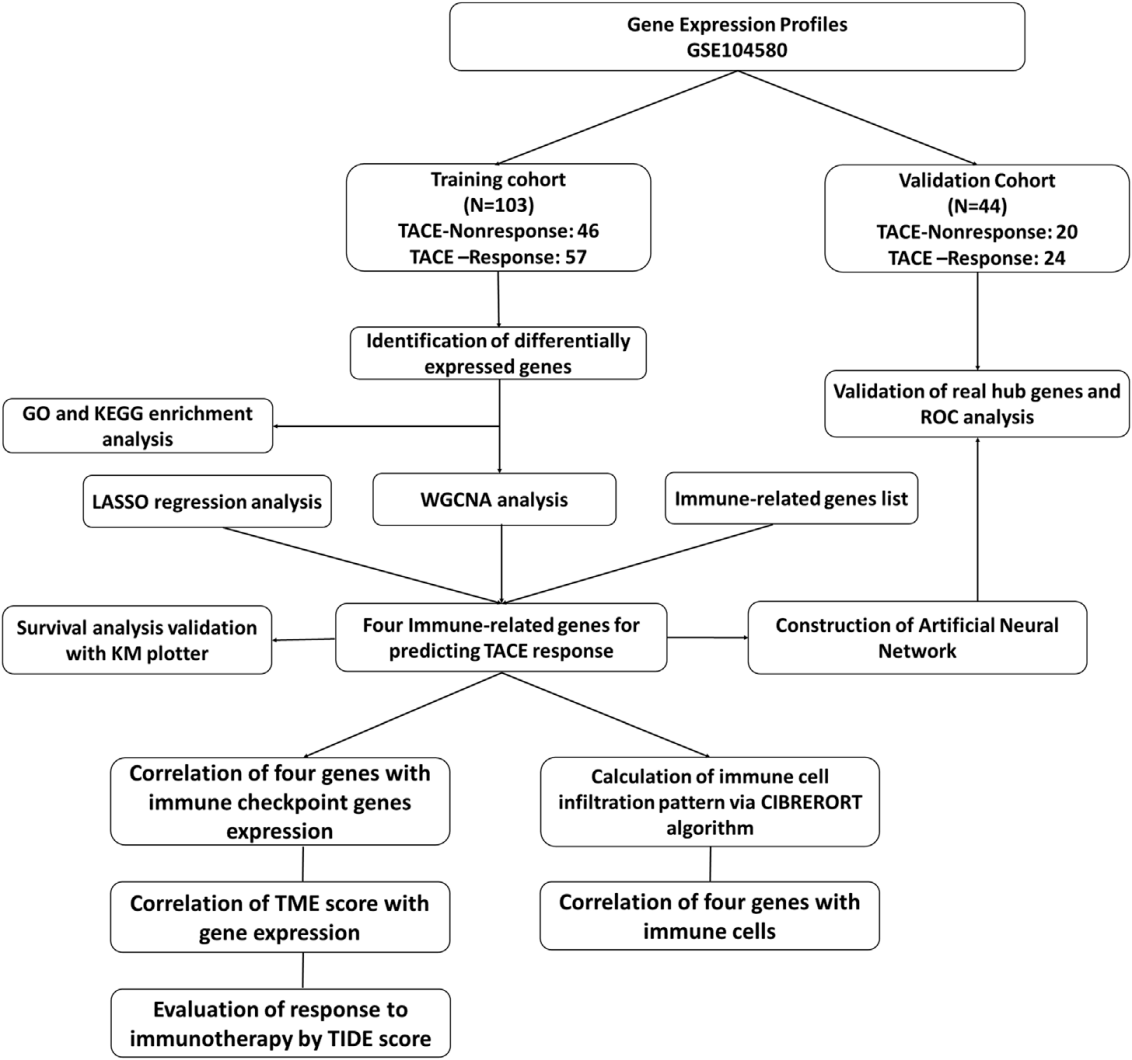
The flowchart of this study.

Gene expression dataset of GSE104580 was downloaded through the GEO database (https://www.ncbi.nlm.nih.gov/geo/). The study design and population was shown in Figure1. The CEL files of all datasets was obtained from the GEO database and then processed with R software (Version 4.0.1). ID conversion was conducted with the R package “org.Hs.eg.db” (v3.13.0). The normalization and background adjustment of expression profiles were conducted with “affy” package.

The dataset comprised of 147 patients with unresectable HCC and no significant baseline liver dysfunction. These treatment-naive patients received TACE as their primary treatment and 81 of them were determined as TACE-Responders and 66 of them were determined as TACE-Nonresponders.

### Identification of differentially expressed genes

The differentially expressed genes (DEGs) between TACE-Nonresponders with TACE-Responders were acquired with Linear Model for Microarray (LIMMA) package to analyze genes expression. A False discovery rate (FDR) value < .05 and |logFC| >1.0 were set to determine the significant differential expression mRNAs in patients with TACE refractoriness. For visualization, the package “ggplot” was used to display the volcano plot of DEGs.

### Gene set enrichment analysis

To clarify the functional roles of DEGs, Gene Ontology (GO) and Kyoto Encyclopedia of Genes and Genomes (KEGG) enrichment analysis were conducted with the Search Tool for the retrieval of Interacting Genes/Proteins database (STRING). The package “clusterProfiler” was utilized to perform GO and KEGG analysis to identify top five types of significantly enriched GO terms (*p* < .05) and pathways (*p* < .05).

### WGCNA analysis

The “WGCNA” package was applied to establish the co-expression network between TACE-Nonresponse and TACE-Response samples. The integrity of data was checked with the “goodSampleGenes” function. Then, pearson’s correlation analysis of all pairs of genes was used to establish an adjacency matrix, which was used to construct a scale-free co-expression network. The adjacency matrix was transformed to a topological overlap matrix (TOM) that could quantify the network connectivity of a gene. Following the calculation of module eigengene (ME) and merging similar modules in the clustering tree based on ME, a hierarchical clustering dendrogram was generated. Gene significance (GS), which was the mediator *p*-value (GS = lg*P*) for each gene, represented the degree of linear correlation between gene expression and clinical states. Module Membership (MM) > .5 and GS > .3 were set as the threshold to screen hub genes in each module.

### LASSO regression analysis and screening for final hub genes

Least absolute shrinkage and selector operation (LASSO) regression with 10-fold cross-validation and penalty was applied to explore important genes from all DEGs further. The list of IRGs was obtained in the Immunology Database and Analysis Portal (ImmPort) database (https://immport.niaid.nih.gov), which timely updates the immunology data and shares the data for immunologic research and provides a list of IRGs for cancer researchers.

Final hub IRGs were determined by overlapping LASSO regression analysis results, IRGs list, and WGCNA analysis results. Venngram was then plotted based on the above results.

### The construction and verification of the artificial neural network model

The training cohort was used to construct the artificial neural network (ANN) model, and validation cohort was used for signature validation. According to the four IRGs selected, an ANN model was constructed by the “neuralnet” R package. The response to TACE classification score was the sum of the multiplied results for hub gene expression and gene weight scores. The discrimination, goodness-of-fit and net clinical benefit of ANN model were assessed by receiver operating characteristic (ROC) curve, calibration curve and decision curve analysis (DCA), respectively.

### Validation of final hub genes expression profiles

Gene expression of final hub IRGs identified in training cohort were verified in validation cohort. Differences of expression between these samples was considered statistically significant with a *p*-value < .05.

### Calculation of immune cell infiltration pattern

In order to compare the differences of infiltrated immune cells between two groups, 66 TACE-Nonresponse and 81 TACE-Response samples were included to evaluate the pattern of immune cells infiltration. CIBERSORT is a computational algorithm for transforming the gene expression matrix to the composition of infiltrating immune cells. A total of 22 kinds of immune cells were estimated. We set the *p*-value at <.01 for statistical significance. The “vioplot” package in R Studio was utilized to visualize the results.

### Correlation between final hub IRGs with tumor microenvironment and immune checkpoints

We used the ESTIMATE algorithm to determine the immune/stromal/Estimate scores and tumor purity of HCC and compared the differences of these scores between high and low expression group stratified with median values by Wilcoxon test. We also explored the potential relationship between IRGs expression with immune cell infiltration in the context of TACE refractoriness, by the “ggpubr” and “ggExtra” package. Then, a lollipop was plotted to visualize the results (Chen et al., 2022). In addition, correlation between hub IRGs with immune checkpoints in HCC was assessed.

### Evaluation of response to immunotherapy by TIDE score

The patient’s response to immune checkpoint inhibitors (ICI) was inferred by the tumor immune dysfunction and exclusion (TIDE) score. The score was compared between TACE-Nonresponse with TACE-Response samples, predicted TACE-Nonresponse with predicted TACE-Response samples, high-expression with low-expression IRG samples. Generally, a lower TIDE score represents better response to immunotherapy.

## Results

### Identification of DEGs and functional enrichment analysis

**FIGURE 2:**
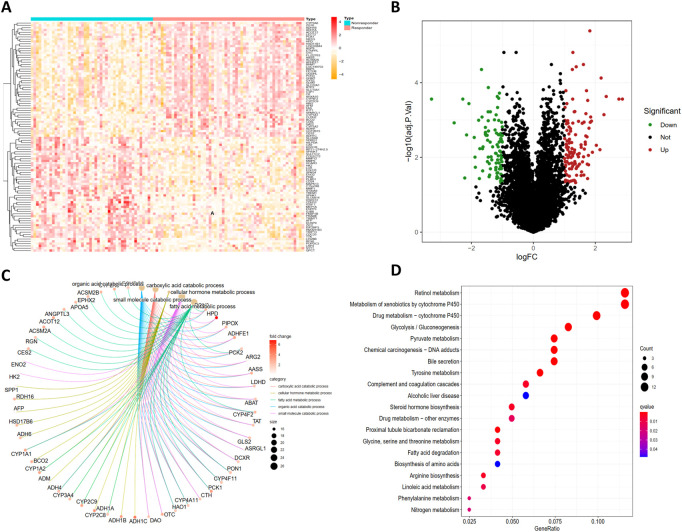
Identification of DEGs and functional enrichment analysis of DEGs. The heatmap **(A)** and volcano plot **(B)** of differentially expressed genes (DEGs) between TACE-Nonresponse and TACE-Response samples, Gene Ontology (GO) **(C)** and Kyoto Encyclopedia of Genes and Genomes (KEGG) **(D)** functional enrichment analysis of DEGs.

A total of 21,653 gene symbols were identified after annotation. We finally identified 127 significantly upregulated and 97 significantly downregulated expressed genes between TACE-Nonresponse and the TACE-Response samples. The heatmap and volcano plot of these DEGs were visualized in Figures 2A, B.

GO analysis demonstrated that identified DEGs were mainly enriched in carboxylic acid catabolic process, cellular hormone metabolic process, fatty acid metabolic process, organic acid catabolic process, and small molecule catabolic process (Figure 2C). KEGG pathway analysis displayed that DEGs are mainly enriched in Retinol metabolism, Metabolism of xenobiotics by cytochrome P450, Drug metabolism - cytochrome P450, Pyruvate metabolism, Tyrosine metabolism (Figure2D).

### Construction of the co-expression network

**FIGURE 3:**
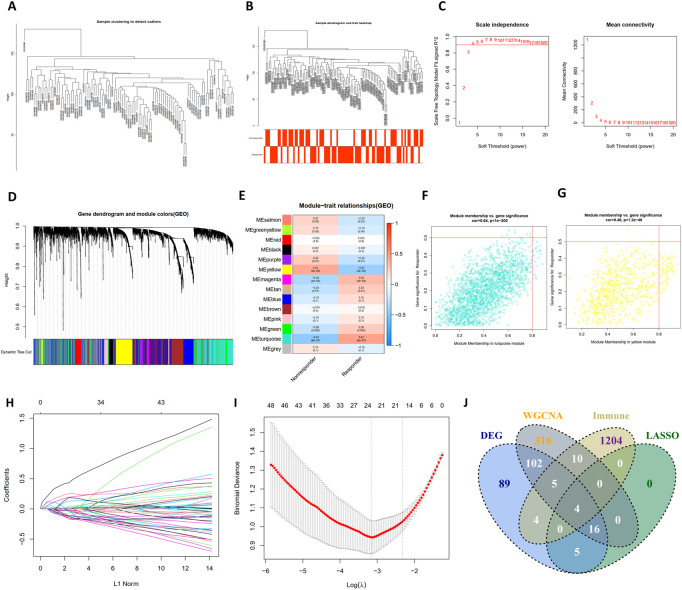
The construction of WGCNA network, LASSO regression analysis and venngram of final hub genes. **(A)** Clustering dendrogram of samples in the dataset GSE104580. **(B)** Clustering dendrogram of 66 TACE-Nonresponders and 81 TACE-Responders samples. **(C)** Analysis of network topology for various soft-thresholding powers. **(D)** Clustering dendrogram of mRNAs, with dissimilarity based on topological overlap, together with assigned module colors. **(E)** The heatmap to show the correlation between module eigengenes and patient response (TACE-Nonresponse and TACE-Response). **(F,G)** The scatter plots of module eigengenes in turquoise module and yellow module. **(H)** The LASSO coefficient profiles of genes, **(I)** The feature selection by LASSO analysis. The *X*-axis shows Log (λ), and the *Y*-axis shows the binomial deviance, **(J)** The venngram of final hub genes.

As illustrated in Figures 3A–D, a total of 14 modules were determined with the dynamic tree cutting method, and each color represented one module. The hub modules were identified through module-trait correlation analysis and eight modules were defined as hub modules (Figure 3E), among which the turquoise module was considered most significant (Figure 3F). Finally, 452 hub genes were identified in the hub modules.

As shown in Figures 3H, I, the log(λ) was set to -3.161178306371 and 24 features were selected by LASSO regression analysis. Then, final hub genes were determined by overlapping the results of DEGs, LASSO regression, WGCNA analysis, and IRGs list, including *S100A9*, *COLEC12*, *IFIT1*, and *TREM1* (Figure 3J). *IFIT1* was significantly downregulated in the TACE refractoriness group, while the remaining three genes were upregulated.

### Construction and evaluation of the ANN model

**FIGURE 4:**
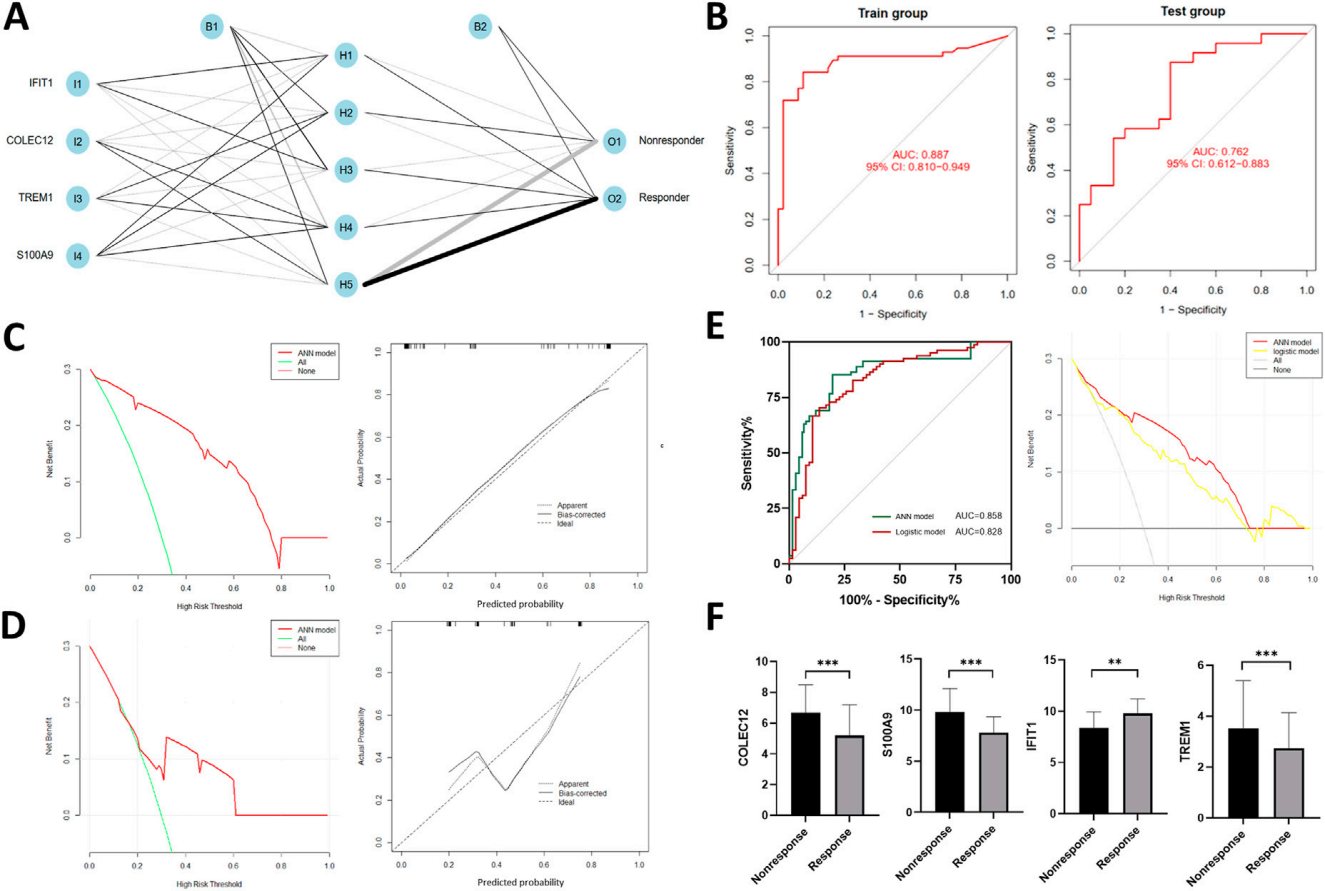
Construction of artificial neural network (ANN) **(A)** and model evaluation. **(B)** Receiver operating characteristics (ROC) curves of the model in training and validation cohort. **(C,D)** Decision curves and calibration curves of ANN model. **(E)** Comparison of discrimination and clinical application between signatures constructed by ANN and traditional linear regression, **(F)** Expression profiles of four IRGs in the validation cohort.

Four IRGs were integrated to construct a neural network in the training group with R package “neuralnet”. The weight of each gene was calculated for optimal differentiation between the TACE-Nonresponse and TACE-Response patients. A prediction model was then established based on the weights of the four IRGs and the neural network. The ANN model contained four input layers, five hidden layers, and two output layers (Figure 4A). Each gene’s neural network weight score was presented in Supplementary Table S1.

The formula of TACE response categorization score of neural network model is: Neura TACE-Response = ∑ (Gene Expression × Neural Network Weight). Prediction accuracy of the model had an AUC of .887 (95% CI, .810–.949) in training cohort and .762 (95% CI, .612–.883) in the validation cohort (Figure 4B), suggesting that the ANN is relatively stable in predicting TACE refractoriness. Besides of ROC analysis, calibration curves and decision curves were plotted and demonstrated that our ANN model performed well in predicting TACE refractoriness (Figures 4C, D).

To compare the prediction efficacy between ANN model and conventional logistic regression model, we performed ROC analysis and plotted DCA curves in the total cohort. The ANN model had better AUC [.857 (95% CI, .789–.908) vs.828 (95% CI, .758–.886)], with more net clinical benefit than logistic regression model (shown in right panel of Figure 4E).

### Validation of final hub genes expression profiles

In the validation cohort, gene expression of *S100A9*, *COLEC12*, *TREM1* were up-regulated while *IFIT1* was down-regulated (Figure 4F), which were consistent with those of training cohort.

### Immune infiltration analysis

**FIGURE 5:**
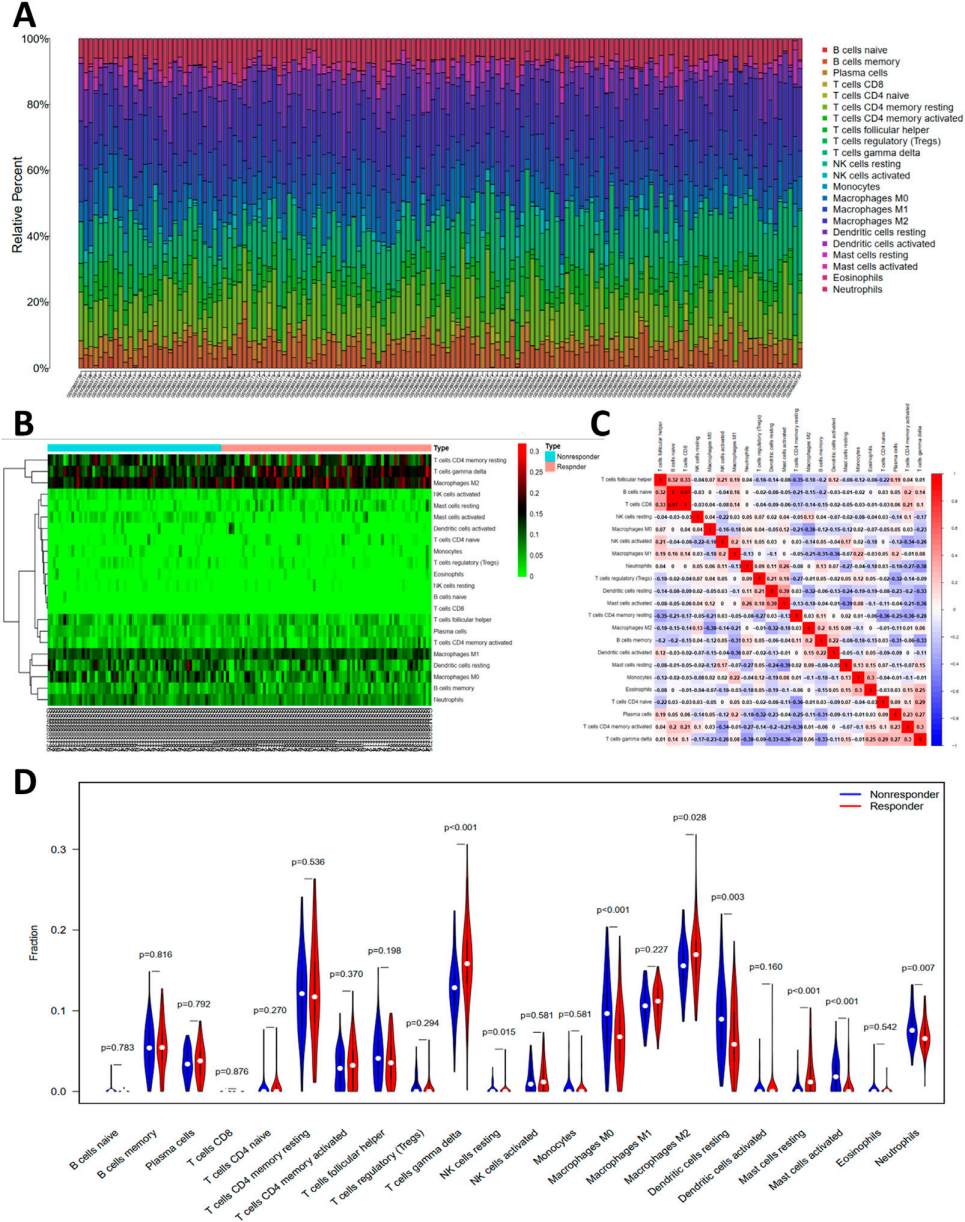
The profiles of immune cell subtype distribution pattern in GSE104580 cohort. **(A)** The bar plot visualizing the relative percent of 22 immune cell in each sample. **(B)** Heatmap of the 22 immune cell proportions in each sample. **(C)** Correlation heatmap of all 22 immune cells. **(D)** Violin plot of all 22 immune cells differentially infiltrated fraction.

The histogram (Figure 5A) showed the general distribution of 22 kinds of immune cells for each sample. Individual differences were observed about the proportions of immune cells between two groups (Figure 5B). The correlation plot (Figure 5C) showed that the proportions of different infiltrated immune cell were correlated to varying degrees. For example, the correlation of T-cells follicular helper cells and T-cells CD4 memory resting is -.35, and the correlation of T-cells CD8 cells and T-cells follicular helper cells is .33. Differences of T-cells gamma delta, NK cells resting, Macrophages M0, Macrophages M2, Dendritic cells resting, Mast cells resting, Mast cells activated, and Neutrophils were significant between two groups (Figure 5D).

### Association between hub IRGs with immune cells infiltration pattern, tumor microenvironment scores, and immune checkpoints

**FIGURE 6:**
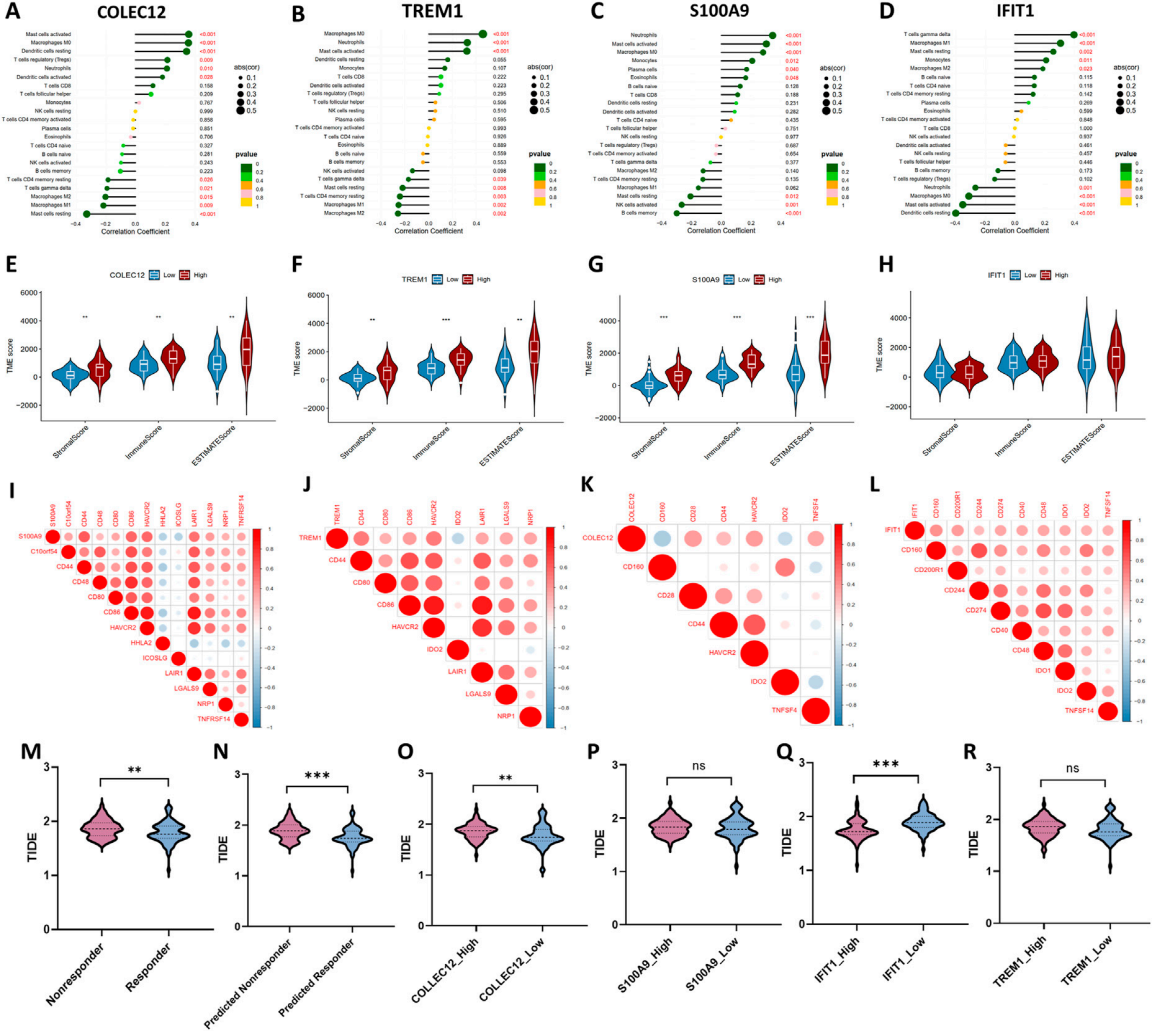
Correlations between four IRGs with infiltrated immune cells **(A–D)**, tumor microenvironment characteristics **(E–H)**, immune checkpoint molecules **(I–L)**, and Tumor immune dysfunction and exclusion (TIDE) scores for evaluating immunotherapy response **(M–R)**.

As illustrated in Figure 6, there were significant correlations between these IRGs and tumor-infiltrating immune cells. Notably, among the 22 types of immune cells, the relative proportion of Macrophages M1, and Mast cells resting were negatively correlated with the *COLEC12*, while the relative proportion of Mast cells activated, Macrophages M0, Dendritic cells resting, T-cells regulatory (Tregs) were positively correlated with *COLEC12* (Figure 6A). The expression of *TREM1* was positively correlated with Mast cells activated, neutrophils, Macrophages M0, while negatively related with Mast cells resting, T-cells CD4 memory resting, Macrophages M1, Macrophages M2 (Figure 6B). The expression level of *S100A9* was negatively correlated with NK cells activated, and B cells memory, while positively correlated with Macrophages M0, Mast cells activated, and Neutrophils (Figure 6C). *IFIT1* expression negatively related with the proportion of neutrophils and Macrophages M0, Mast cells activated, and Dendritic cells resting, while positively correlated with Mast cells resting, Macrophages M1, and T-cells gamma delta (Figure 6D).

Moreover, the ESTIMATE algorithm was utilized to estimate the immune score, stromal score, and tumor purity, which represents the tumor environment. These scores all increased in high-expression group of *COLEC12*, *TREM1* and *S100A9*, while the corresponding tumor purity decreased (Figures 6E–H), which further confirmed the roles of these IRGs in regulating tumor microenvironment.

In addition, the expression levels of 45 potentially targetable immune checkpoint genes were compared between the subgroups. Patients in the high-expression *S100A9* group had significantly increased *C10orf54*, *CD44*, *CD48*, *CD80*, *HAVCR2*, *LAIR1*, *LGALS9*, *NRP1*, *TNFRSF14* and decreased *HHLA2,* and *ICOSLG* (Figure 6I). The expression of *TREM1* positively correlated with *CD44*, *CD80*, *CD86*, *HAVCR2*, *LAIR1*, *LGALS9*, and *NRP1*, while negatively correlated with *HHLA2*, and *ICOSLG* (Figure 6J). *COLEC12* expression positively correlated with *CD28*, *CD44*, *HAVCR2*, and *TNFSF4*, while negatively correlated with *CD160* and *ID O 2* (Figure 6K). *IFIT1* expression positively correlated with *CD160*, *CD200R1*, *CD244*, *CD274*, *CD40*, *CD48*, *ID O 1*, *ID O 2*, and *TNFSF4* (Figure 6L).

### Response to immunotherapy

To explore the effect of the four IRGs on immunotherapy, the TIDE algorithm was used. As the results demonstrated, TIDE score was higher in TACE-Nonresponse group than TACE-Response group (1.87 ± .16 vs. 1.79 ± .20, *p* = .011), indicating that patients with TACE refractoriness might have worse response to immunotherapy. The predicted TACE-Nonresponder also had a higher TIDE score than predicted TACE-Responder (1.89 ± .16 vs. 1.76 ± .18, *p* < .001) (Figures 6M–N). In addition, higher expression levels of *COLEC12*, *S100A9*, *TREM1* and lower expression level of *IFIT1* were associated with poor response to immunotherapy in patients with HCC, suggesting that these IRGs probably affect the efficacy of ICI in HCC patients (Figures 6O–R).

## Discussion

Although TACE is the standard treatment option for intermediate-stage HCC, the response to TACE is quite variable among HCC patients. Currently, it is essential to identify early biomarkers of TACE refractoriness and select individualized treatment strategies. In the present study, differential analysis was first performed to screen out 224 DEGs. Then, LASSO regression analysis was performed to further select 25 genes from all DEGs. A total of 152 genes were identified through WGCNA analysis. Four hub genes were finally determined by the intersection of LASSO analysis results, WGCNA results and immune-related genes list. Based on the four genes, the ANN model was constructed and validated. ROC analysis exhibited satisfactory predictive value of ANN model, with .887 in training cohort and .762 in validation cohort. Correlation between four hub genes with immune cells and immune checkpoint genes was analyzed and we found that four IRGs were associated with infiltrated immune cells and correlated with different immune checkpoint molecules in various degrees. Patients with TACE-Response, predicted TACE-Response, lower expression of *COLEC12*, *S100A9*, *TREM1* and higher expression of *IFIT1* had lower TIDE score, indicating better response to immunotherapy.

About the four IRGs involved in the signature, we found that all of them were related with tumor immunity. Interferon-induced protein with tetratricopeptide repeats (IFIT) genes, as prominent interferon-stimulated gene, consists of *IFIT1*, *IFIT2*, *IFIT3* and *IFIT5* (Pidugu et al., 2019a). Pidugu et al. (2019b) revealed the role of *IFIT1* and *IFIT3* in driving OSCC progression and metastasis. Liu et al. (2021) demonstrated that *IFIT1* and *IFIT3* expression could modulate cell migration and metastasis in HCC patients. *IFIT1* or *IFIT3* silencing reduced the expression of IL-17 and IL-1β, and attenuated the migration capability of HCC cells. Yap et al. (2020) suggested that *ANXA1* plays a regulatory role in RIG-I signaling and cell death in A549 lung epithelial cells, while silencing *IFIT1* could inhibit RIG-I-induced cell death. Interestingly, lower expression of *IFIT1* was identified to be related with TACE refractoriness in our study. In addition, higher expression group of *IFIT1* was related to longer overall survival. *COLEC12* is a member of the C-type Lectin receptor family, affecting the adaptive immune response through the carbohydrate-recognition domain *via* the recognition of the complex glycan structures of pathogens. Huang et al. (2021) demonstrated that higher expression of *COLEC12* was correlated with shorter OS and involved in immunosuppression for HCC patients. Zhang et al. (2021) constructed an immune-related signature in which higher expression of *COLEC12* was related with shorter survival and more sensitive to immune checkpoint inhibitors (ICIs) treatment.

Triggering receptors expressed on myeloid cells 1 (*TREM-1*), as a member of the TREM family (Sun et al., 2020), is an inflammatory modulator. Duan et al. (2015) claimed that higher expression of *TREM1* correlated significantly with increased recurrence and poorer survival in HCC patients. Chen et al. (2021) found that downregulating *TREM1* expression in macrophages shift M2 macrophages towards a M1 phenotype by inhibiting PI3K/AKT signaling, inhibiting migration and invasion of live cancer. *S100A9* belongs to the S100 family of calcium-binding proteins and is over-expressed in hepatocellular carcinoma (HCC). As indicted by Wu et al. (2013), *S100A9* could promote the proliferation and invasion of HepG2 HCC cells *via* activating the MAPK signaling pathway. Higher serum *S100A9* is reported to be associated with worse outcome in HCC patients receiving resection (Meng et al., 2019). Through immunohistochemistry analysis of tissues from HCC patients, Liao et al. (2021) discovered that the infiltration of *S100A9*+ cells in both tumor and non-tumor tissues could predict poor OS and a higher recurrence risk.

Many previous studies have attempted to construct the immune-related, apoptosis-related, Cuproptosis-related, or Ferroptosis-related gene signatures to predict cancer prognosis and correlate these genes with immune cells infiltration patterns (Wang et al., 2022b; Luo et al., 2022; Ma et al., 2022; Zhang et al., 2022). There may be many potential mechanisms of TACE refractoriness. According to the study by Dong et al. (2018), hypoxia-induced angiogenesis is the potential underlying mechanism of TACE failure. Cheng et al. (2022) also found that hypoxia-related genes are potential biomarkers for patients with refractory TACE and patients at high hypoxic risk have more active immune microenvironment). Our study screened DEGs between TACE-refractory with TACE-response HCC patients and constructed a signature from the immunological perspective. We also investigated the correlation of signature genes with tumor microenvironment scores, infiltrated immune cells and immune checkpoint molecules. In essence, these are different means of dimension reduction that could more specifically reflect tumor response to TACE procedures from respective pathways, with certain selection bias.

The prognosis of HCC patients treated with TACE varies due to highly heterogeneous tumor biological characteristics, not always positively impacting survival (Ringelhan et al., 2018). In addition, repeated TACE is often recommended because it is sometimes difficult to achieve a satisfactory tumor response with a single session (Chen et al., 2022). However, we should also be acknowledged that high frequency and number of TACE operations may cause liver function impairment and increasing treatment-related adverse events. Though, there is no clear consensus on the definition of TACE refractoriness. The concept is put forward to take full advantage of TACE treatment while reducing repeat or ineffective TACE induced liver function damage and other complications as far as possible.

There are still several limitations that should also be noted. First, this is a small sample size retrospective study, and the training and validation cohort were split from the same dataset. Further investigation needed more samples from various institutions for verification. Second, the related clinical and imaging characteristics were unavailable from the dataset, failing to integrate these important variables into the model. Third, although with better prediction accuracy, the interpretability of ANN model used in our study is not better than the conventional algorithms, which can provide specific possibility value for individuals. Last, the experiments confirmation is lacking and further molecular biology studies would be supportive.

## Conclusion

In this study, immune-related genes of *S100A9*, *COLEC12*, *TREM1,* and *IFIT1* were identified to be associated with TACE refractoriness and integrated to establish a machine-learning based signature with satisfactory performance, which may have the potential to be the immunotherapeutic targets for TACE refractory HCC patients. We anticipate this tool can provide guidance for clinicians to make decisions for HCC patients.

## Data Availability

Publicly available datasets were analyzed in this study. This data can be found here: https://www.ncbi.nlm.nih.gov/geo/query/acc.cgi?acc&equals;GSE104580.
